# Nano-plasmonic near field phase matching of attosecond pulses

**DOI:** 10.1038/s41598-017-06491-7

**Published:** 2017-07-25

**Authors:** Tahir Shaaran, Rana Nicolas, Bianca Iwan, Milutin Kovacev, Hamed Merdji

**Affiliations:** 1 LIDYL, CEA, CNRS, Université Paris-Saclay, CEA Saclay, 91191 Gif-sur-Yvette France; 2 Leibniz Universität Hannover, Institut für Quantenoptik, Welfengarten 1, D-30167 Hannover, Germany; 3 QUEST, Centre for Quantum Engineering and Space-Time Research, Hannover, Germany

## Abstract

Nano-structures excited by light can enhance locally the electric field when tuned to plasmonic resonances. This phenomenon can be used to boost non-linear processes such as harmonic generation in crystals or in gases, Raman excitation, and four wave mixing. Here we present a theoretical investigation of the near-field phase matching of attosecond pulses emitted by high-order harmonic generation (HHG) of an atom immersed in a multi-cycle femtosecond infrared laser field and a spatially inhomogeneous plasmonic field. We demonstrate that the spatial inhomogeneity factor of the plasmonic field strongly affects the electron trajectory and recombination time which can be used to control the attosecond emission. For further insight into the plasmonic field effect, we monitor the phase of each quantum path as a function of the inhomogeneity strength. Moreover, we investigate the attosecond emission as a function of near-field phase matching effects. This is achieved by calculating the coherent field superposition of attosecond pulses emitted from various intensities or field inhomogeneities. Finally, far-field and near-field phase matching effects are combined to modulate the harmonic spectral phase towards the emission of a single attosecond pulse.

## Introduction

The high-order harmonics generation (HHG) process^1, 2^ is a unique source of coherent radiation in the ultraviolet to extreme ultraviolet (XUV) spectral domain. Apart from its numerous applications in various areas of science^3^, HHG has been employed for generating ultrashort pulses down to the single attosecond pulse regime^4^. The harmonic emission can be described by the semi-classical three step model^5^. An electron leaves the atom into the continuum by tunneling though the atomic potential barrier which is modified by the incident laser electric field. It subsequently propagates in the continuum and is driven back by the laser field towards its parent ion. In the last step, it recombines with the core, leading to the emission of energetic photons. The generation process in gases requires driving intensities in the 10^13^–10^15^ W/cm^2^ range, requiring the use of expensive amplified laser chains.

**Figure 1:**
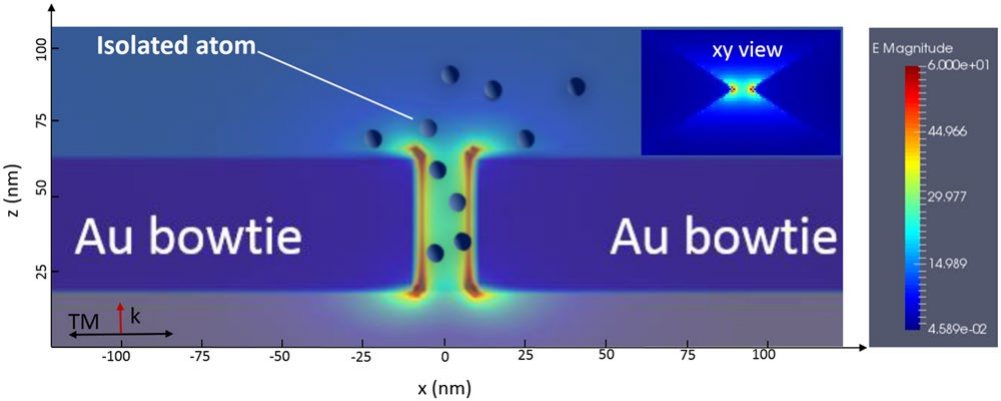
XZ plane representation of the plasmonic field enhancement from two gold nano-antennas. The XY representation is shown as an inset. The field enhancement is calculated using FTDT approach at a laser wavelength of 825 nm. Atoms are sketched as spheres immersed in the field enhanced by the two antennas. The field is polarized along the *TM* axis and propagates along the *k* vector.

An interesting proposition to obtaining XUV radiation at extremely high repetition rate without heavy and costly laser pumping is to use plasmonic field amplification of a femtosecond oscillator in resonant gold nano-antennas^6– 9^. The external femtosecond low intensity laser pulse couples to the plasmon mode of the nanostructure and induces a collective oscillation of free charges within a tightly localized region. The free charges redistribute the electric field in the vicinity of the nanostructure to form a spot of highly enhanced electric field as shown in Fig. 1 for gold nano-antennas. The enhanced field largely depends on the geometrical shape of the metallic nanostructure. Plasmonic effects are strongly localized and can be used to concentrate light or to boost non-linear phenomena like third harmonic generation for example﻿﻿﻿^10–15^. However, the experimental feasibility of HHG in gases assisted by plasmonic field enhancement^6, 7^ was challenged in successive publications^16–19^. The main argument is that the small and strongly localized excitation volume does not allow an efficient buildup of coherent harmonic radiation in the enhanced laser field. Instead, the measured signal reported in refs 6 and 7 is dominated by incoherent fluorescence emission as recently reported by Han *et al*.^20^.

The controversy on the feasibility of plasmon-enhanced HHG in gases shows that the observation of coherent radiation poses a difficult experimental challenge due to the small emission volume^6–9^ and the dominance of atomic plasma lines^16–22^. However, for an experiment using the bowtie geometry^8^ the XUV emission could be interpreted as an almost equal level of plasma and harmonic radiation. But the thermal damage of the gold nanostructures limits the applicability of the proposal^22^. Future experiments will need to solve this issue by improving the nanostructure design, and material choices, plasmonic excitation volume and homogeneity, as well as laser parameters. In this this quest, HHG in graphene assisted by plasmonic field amplification has been theoretically proposed^23^. Experimentally, a recent outcome is the unambiguous observation of HHG in crystals assisted by plasmonic field amplification^20, 24^. The high density of atoms in solid density media promotes the coherent build-up of the harmonic radiation compared to gases. This confirms that strong field phenomena can be effectively boosted using local field enhancement and that the theoretical concept of plasmon-enhanced HHG in gases stands. Our theoretical concept can be transferred to other materials, like solids, using a valid model of HHG in solids. This is however out of the scope of this letter. There exists a considerable body of theoretical work investigating HHG from gaseous media driven by spatially inhomogeneous field^25–38^. This offers exciting perspectives in the field of plasmon-enhanced strong-field physics and attophysics^39^. This gives the possibility for example to shape or enhance HHG spatial or spectral properties by spatially rearranging the nanostructures. Indeed, the locally enhanced field has a distinct spatial dependence, which could produce an enormous extension to the HHG cutoff ^25^.

## Results and Discussions

In this letter, we investigate the attosecond emission from atoms subjected to a strong laser field that is enhanced and modulated by a local plasmonic field gradient. For that purpose, we calculate how the harmonic spectral phase from atoms under various plasmonic field strength varies. The strong field approximation (SFA)^40^ is applied to investigate the phase and the cutoff behavior of the high order harmonics generated by the spatially inhomogeneous field^25^. A detailed analysis of the individual electron trajectories in comparison to their classical counterparts demonstrates their contributions to the HHG cutoff. In addition, we extend our model to include the fact that not all atoms inside the plasmonic field experience the same intensity and inhomogeneity but a gradient. Here, we use the field gradient to achieve near field phase matching of attosecond pulses. Similarly to the pioneering work of Antoine *et al*., we found that the phase of the harmonic comb has to be controlled to coherently build the attosecond emission^41, 42^.

Our approach uses a transition amplitude for HHG calculated under the SFA formalism. The spatial dependence of the laser electric field is perturbative and linear with respect to the position (for details see ref. 28). Therefore, the locally enhanced field can be approximated as

1 $$E(t{,}x)\simeq E(t)(1+{\epsilon }x)$$

where ε ≪ 1 is a parameter that we define as the inhomogeneity field factor. Depending on both the geometrical and material characteristics of the nanostructure this parameter reflects the spatial decay of the plasmonic near-field. In the linear model used, the unit of ε is inverse length. It corresponds to the first term of the actual field of the plasmonic nanostructure. Figure 1 shows finite difference time domain (FDTD) calculations of the plasmonic field enhancement in the center of a gold bowtie nanostructure under a strong laser field. Neon atoms are present in the 20 nm wide gap and the vicinity of the bowtie. As shown in Fig. 1, the small gap shows a very sharp gradient in the electric field, varying from 30 to 60 in field enhancement (i.e., 900 to 3600 in intensity enhancement respectively). These are common values for bowtie nano-antennas and other typical plasmonic structures^6, 8, 16–19, 22^. The atoms inside the gap will interact with various inhomogeneous field factors and are thus subjected to stronger field amplification close to the gold bowties and weaker field amplification as the distance from the bowtie gap and edges increases (in Fig. 1 the plasmonic field magnitude is represented in color code). Our approach consists first of calculating the total electric field for a given value of *ε*. In Eq. (1), the approximation corresponds to the first term of the actual field of a spherical plasmonic nanostructure^35^. Then, by incorporating Eq. (1) in the Lewenstein model^40^, the transition amplitude describes the HHG in a spatially inhomogeneous field under the SFA approximation. We assume here that the electric field is linearly polarized along the z-axis (see Fig. 1). To get a better insight into the nonhomogeneous case, we employ a monochromatic field with *E*(*t*) = *E*_*0*_ *sin*(*ωt*)*e*_*z*_, where *e*_*z*_ is the polarization vector along the *z*-axis. The transition amplitude is computed either using the saddle-point method or numerically^43, 44^. The former method allows us to calculate the contributions of the individual quantum orbits to the cutoff and yield of the HHG, as well as the harmonic phase and time profile of the spectra.

**Figure 2:**
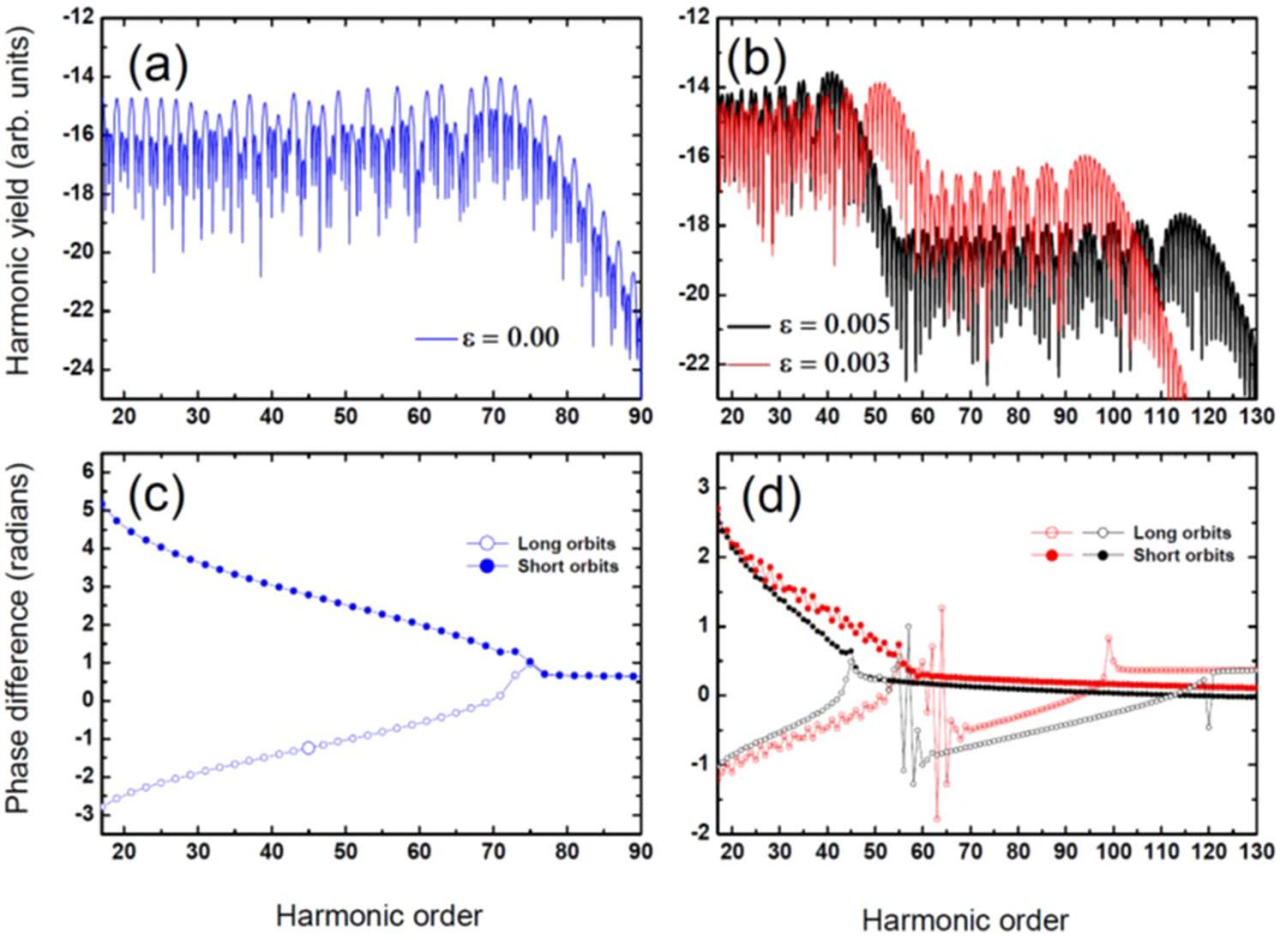
High-order harmonic spectra for a neon atom (*I*_*p*_ = 0.793 a.u.) interacting with a monochromatic field of frequency *ω* = 0.055 a.u. (wavelength of 825 nm) and intensity *I* = 4 10^14^ *W/cm²*. In panel (a) the blue curve depicts the case with *ε* = 0, whereas panel (b) the red and black curves show the cases with *ε* = 0.003 and *ε* = 0.005 inhomogeneity factors respectively. The yields are represented in logarithmic scale. Panels (c) and (d) present the calculated phase difference for the homogeneous case (blue curve) and for the inhomogeneous cases for *ε* = 0.003 (red) and *ε* = 0.005 (black) inhomogeneity factors.

We investigate here how the individual electronic trajectories are affected by the plasmonic field strength, thus modifying the HHG spectral and temporal properties. We restrict ourselves to the solutions of the saddle points of the shortest trajectory pair of our defined monochromatic field. Each of these pairs consists of short and long trajectories. Classically, they correspond to those electrons that most probably ionize at the maximum of the electric field and return to their parent ions at the electric field zero crossing at about half an optical cycle later. Figure 2a presents HHG spectra for a neon atom exposed to a 825 nm wavelength laser radiation at an intensity of *I* = *4*. *10* ^*14*^ *W/cm* ^*2*^ and with *ε* = *0*. Note that the intensity under consideration is the plasmon-enhanced one. As expected, the harmonic spectrum presents a broad plateau of almost constant conversion efficiency, ending with a sharp cutoff for harmonic energies higher than ≃*I*_*p*_ + *3*.*2U*_*p*_ ≃ *69* *ω*, where *U*_*p*_ is the ponderomotive energy. In Fig. 2b, the spectra show the cases for *ε* = *0*.*003* and *ε* = *0*.*005*. The harmonic spectra contain the first plateau, followed by a cutoff, and then the second plateau, ending with a very sharp cutoff. The yields of the first plateaus are almost 2 and 4 orders of magnitude higher than the second plateaus for *ε* = *0*.*003* and *ε* = *0*.*005* respectively.

For the case with *ε* = *0*.*003*, the first and the second cutoffs are located at frequencies around *51* *ω* and *95* *ω*, respectively. For *ε* = *0*.*005*, the first cutoff decreases to *41* *ω* and the second one extends to *115* *ω*. This behavior can be understood by calculating the extension of the first and the second plateaus as a function of the field inhomogeneity strength using the appropriate saddle point solutions. The first plateau and cutoff arise when an electron tunnel-ionizes at the first half of the optical cycle, while the second plateau and cutoff represent the electron tunneling at the second half of the cycle. As a consequence, electrons ejected at the first or second half of the driving laser optical cycle will, respectively, be decelerated or accelerated by the plasmonic field leading to the observed two cutoffs and plateau behaviors. The extension of the first and the second plateau scales, respectively, inversely or proportional with the plasmonic field inhomogeneity factor *ε*. This behavior is similarly to the case where high harmonics are generated with coherently superimposed two-color laser fields^45^. Indeed, we will have either a constructive or a destructive coherent sum of the driving and plasmonic fields, which will modulate the electron kinetic energy and thus the plateau extension. The lower harmonic yield of the second plateau is associated with the fact that those electrons that are further accelerated will experience a higher decoherence (or quantum diffusion) during their excursion in the continuum. Another consequence is that the inhomogeneity of the field breaks the symmetry. Thus, the electron tunnel-ionizes at each half-cycle of the laser field and will have different ionization probabilities and recollision times. The symmetry breaking allows the observation of both even and odd harmonics over the whole range of the spectra (see Fig. 2b), whereas for the homogeneous case only odd harmonics are observed (Fig. 2a).

Next, we perform a detailed analysis of the spectral phase difference between successive harmonics in the spectra to investigate the behavior of the HHG time structure modulated by the plasmonic field. For this purpose, we show in Fig. 2c and d the spectral phase difference by considering both short and long trajectories for the cases with *ε* = *0* and *ε* = *0*.*003*, *ε* = *0*.*005* respectively. For all cases, the phase difference for the short trajectories decreases as a function of the harmonic order along the first plateau whereas it increases for the long trajectories. For the homogenous case (Fig. 2c), which has a single plateau followed by a sharp cutoff, the long and short trajectories merge in the cutoff region exhibiting the same constant phase difference. This behavior is well known as the zero-chirp in the attosecond emission and has been used experimentally for the generation of isolated attosecond pulses^46^. For the inhomogeneous case (Fig. 2d), the attosecond chirp in the first plateau region is not as smooth as in the homogeneous case. This is even more pronounced for an inhomogeneity factor of *ε* = *0*.*003* which corresponds to a smaller field enhancement. After initially merging around the first cutoff, i.e, in the second plateau region, the long and short trajectories diverge from each other. The short trajectories have a constant phase difference along the rest of the spectrum, while the long trajectories, after experiencing a phase jump around the first cutoff, will have almost linear phase increase towards the short trajectories. Then, we reach the second cutoff where the phase difference between consecutive harmonics becomes constant. This reveals a complex behavior of the electron dynamics for both short and long trajectories which will be investigated in the time domain in the next section. Nonetheless, part of the spectrum where the phase difference between consecutive harmonics is constant, which corresponds to a constant attosecond chirp, can be selected. This is mandatory to obtain a regular attosecond pulse train emission as Antoine *et al*. have reported in their calculations^42^ and which was measured later by Mairesse *et al*.^47^. Our analysis shows that the harmonic spectrum of atoms subjected to inhomogeneous plasmonic fields has –at least in parts- the potential for attosecond pulse emission.

**Figure 3:**
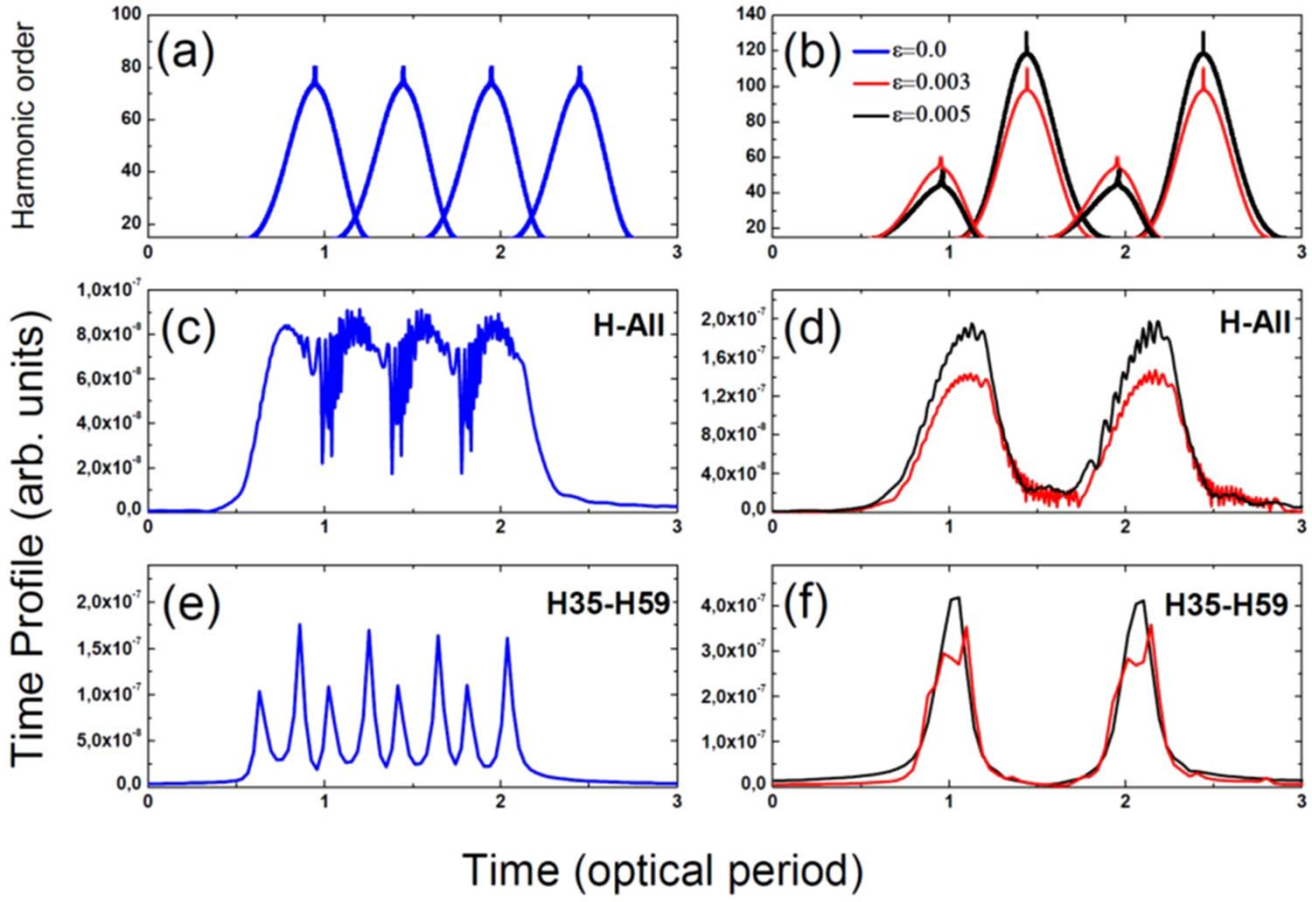
Time profile of high-order harmonic spectra using same parameters as in Fig. 2. Panels (a) and (b) show the harmonic order as a function of the recombination times of the electron. In panels, (c) and (d) the train of pulses emitted from all the harmonics presented in Fig. 2 are reported, while panels (e) and (f) present the train of the pulses emitted from 35^st^ to 59^st^ harmonics. Blue, red and black color curves depict the cases with *ε* = 0, *ε* = 0.003 and *ε* = 0.005, respectively.

In the next step, we characterize the temporal profile of the HHG emission as a function of the field inhomogeneity factor. First, panels (a) and (b) of Fig. 3 show the harmonic order as a function of the real parts of the recombination times of the electron with its parent ion without (a) and with (b) field inhomogeneity. Details about the physical meaning of this representation can be found in refs 40 and 47. In all cases, short and long trajectories are merging in the cutoff. However, we clearly see that the electron dynamics is affected even for low values of the field inhomogeneity factor. In Fig. 3b, we observe that the cutoff is extended only for the second (T = 1.4 optical period) and the last (T = 2.4 optical period) electron excursion whereas it is reduced at T = 0.9 and T = 1.9 optical cycles. Due to the coherence of the HHG process, this control of the electron trajectories will shape the attosecond emission. We calculate now the time profile of the harmonic emission. For that purpose, we use the HHG amplitudes and spectral phases calculated and shown in Fig. 2. By applying an inverse Fourier transform to those spectra, we obtain the time profile of the harmonic emission. For the homogeneous case (panel 3c), we see an almost continuous emission which is due to the attosecond chirp. For the inhomogeneous case (panel 3d), one burst is emitted per cycle. The influence of the plasmonic field on the HHG emission is quite consistent and even applies for a small degree of inhomogeneity (*ε* = *0*.*003*). Furthermore, the attosecond emission in the inhomogeneous case exhibits less noise than in the homogenous case. This noise originates from interferences between different trajectories. As less trajectories contribute to higher harmonics, those interferences will be less pronounced. As a general trend, when *ε* increases, the noise becomes less pronounced. The dominant peak of the time profile increases as a function of the field inhomogeneity and, for *ε* = *0*.*005* is 2 times higher than in the homogeneous case.

We restrict now our analysis to harmonics between the 35^th^ and 59^th^ orders (Fig. 3, panels e and f). Those harmonics mainly belong to the first plateau and have the highest yield. For *ε* = 0 (Fig. 3e), the train of pulses consists of 4 peaks per cycle. The small and large peaks correspond to the recombination times of the short and the long trajectories, respectively. For *ε* = 0.003 and *ε* = 0.005 (Fig. 3f), we observe a single predominant peak per optical cycle. The peak in the case of *ε* = 0.003 is a bit smaller and wider than the peak observed for *ε* = 0.005. Peaks corresponding to attosecond emissions from the second half of the optical cycle disappear when the field inhomogeneity increases. When the long and short trajectories merge, one predominant attosecond pulse is emitted. The height of this peak increases as *ε* become larger (Fig. 3f).

**Figure 4:**
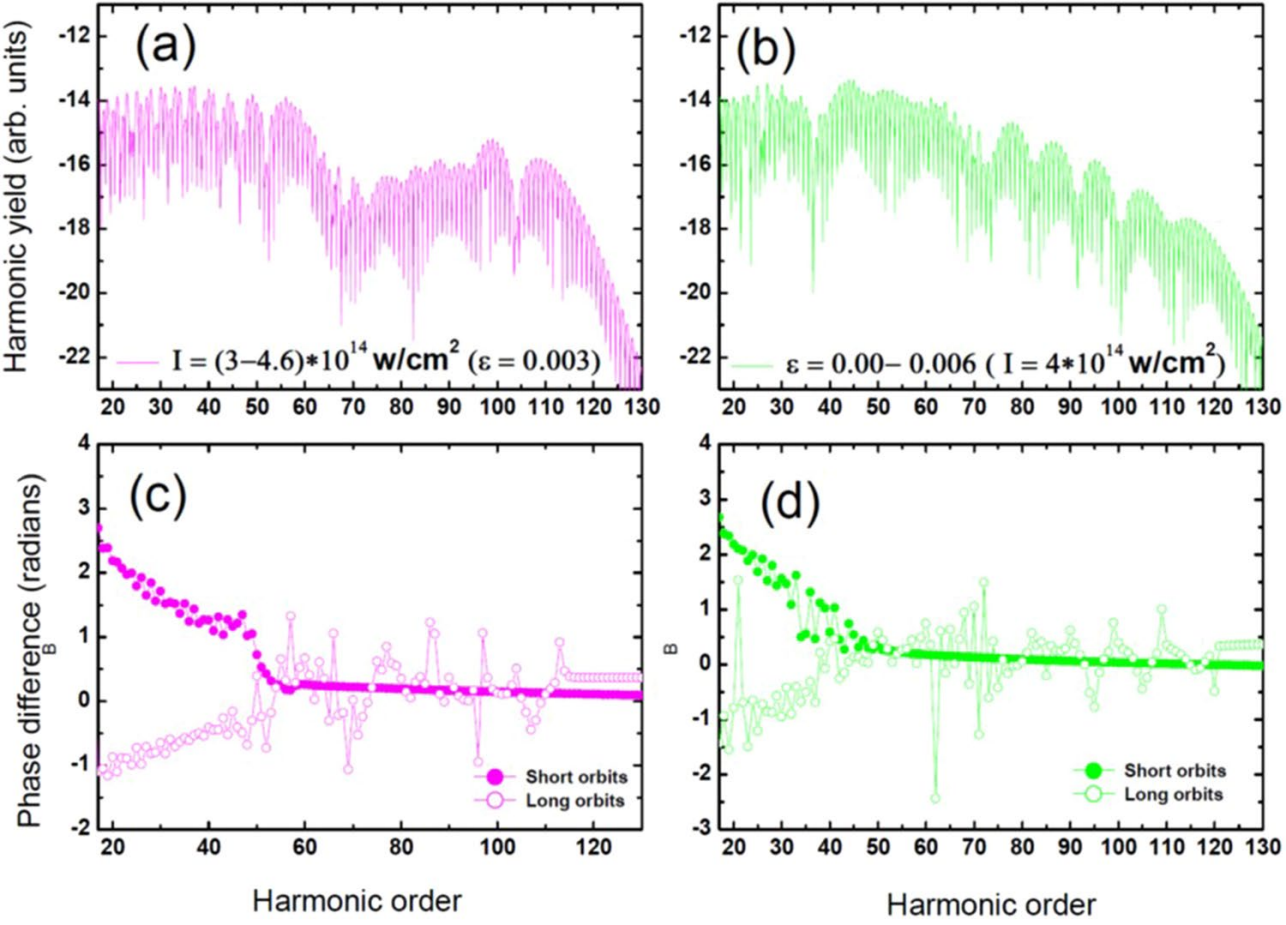
Near field phase matching High-order harmonic spectra in amplitude (**a** and **b**) (in logarithmic scale) for a neon atom exposed to a monochromatic field of frequency *ω* = 0.055 a.u. (wavelength of 825 nm). In panel (a) the intensity varies from *I* = 3.10^14^ W/cm² to *I* = 4.*6*.10^14^ W/cm² while *ε* = 0.003. In panel (b) the intensity is constant (*I* = 4.10^14^ W/cm²) while intensity range from *ε* = 0 to 0.006. For both above cases, the phase difference is calculated both for short (closed circles) and long orbits (open circles), panels (c and d). The yield of total HHG spectra is calculated as the coherent sum of all spectra computed at each inhomogeneity strength with steps of 0.001.

Up to now, we have considered a single field inhomogeneity value at a time. However, on the microscopic level, the individual atoms in the interaction region do not experience the same plasmon-enhanced intensity and field inhomogeneity. Rather, as shown in Fig. 1, individual atoms experience individual intensities and field inhomogeneity factors inside the entire plasmonic excitation volume. The generated high harmonic field thus manifests as the response of an ensemble of atoms with differing electron dynamics and emission processes. These variations in emission dynamics have to be taken into account to correctly describe the resulting HHG field. Therefore, we extend our model by adding coherently the HHG emission from atoms exposed to various field enhancement factors to achieve “near field phase matching”. In Fig. 4a, we keep the field inhomogeneity constant (*ε* = 0.003) and vary the intensity from *3*.*10* ^*14*^ *W/cm²* to *4*.*6*.*10* ^*14*^ *W/cm²*. Then we add coherently all the plasmon-enhanced transition amplitudes. In Fig. 4b, we vary the field inhomogeneity factor from *ε* = 0 to *ε* = *0*.*006* while we keep the intensity constant (*I* = *4*.*10* ^*14*^ *W/cm²*). In the former case, the general shape of the spectra remains unchanged (see Fig. 2b). Due to the increase in intensity, only the position of the second cutoff is extended towards higher harmonics. For the latter case, there is a distortion in the general shape of the spectra and one cannot clearly identify the first and the second plateaus anymore. After the first plateau, the spectra decays until it reaches the second cutoff. In comparison to the case with no near field phase matching, i.e. when (*I* = *4*.*10* ^*14*^ *W/cm²*) and (*ε* = *0*.*005*) in Fig. 2b, the cutoffs of the spectra extend towards higher harmonics. The highest harmonics (above 130) obtained here are associated with the highest field inhomogeneity factor of *ε* = *0*.*006*. Note that the near field phase matching does not distort the harmonic orders and we obtain both even and odd harmonics. For both cases, the phase difference (see Fig. 4c and Fig. 4d) between consecutive harmonics for short and long orbits is irregular up to harmonic *50*. Then, the two trajectories merge in the cutoff with a chaotic behavior for the long trajectory and a more regular one for the short one. This reflects a collective electron dynamics effect where only short trajectory high kinetic energy electrons are preserved from phase distortions.

**Figure 5:**
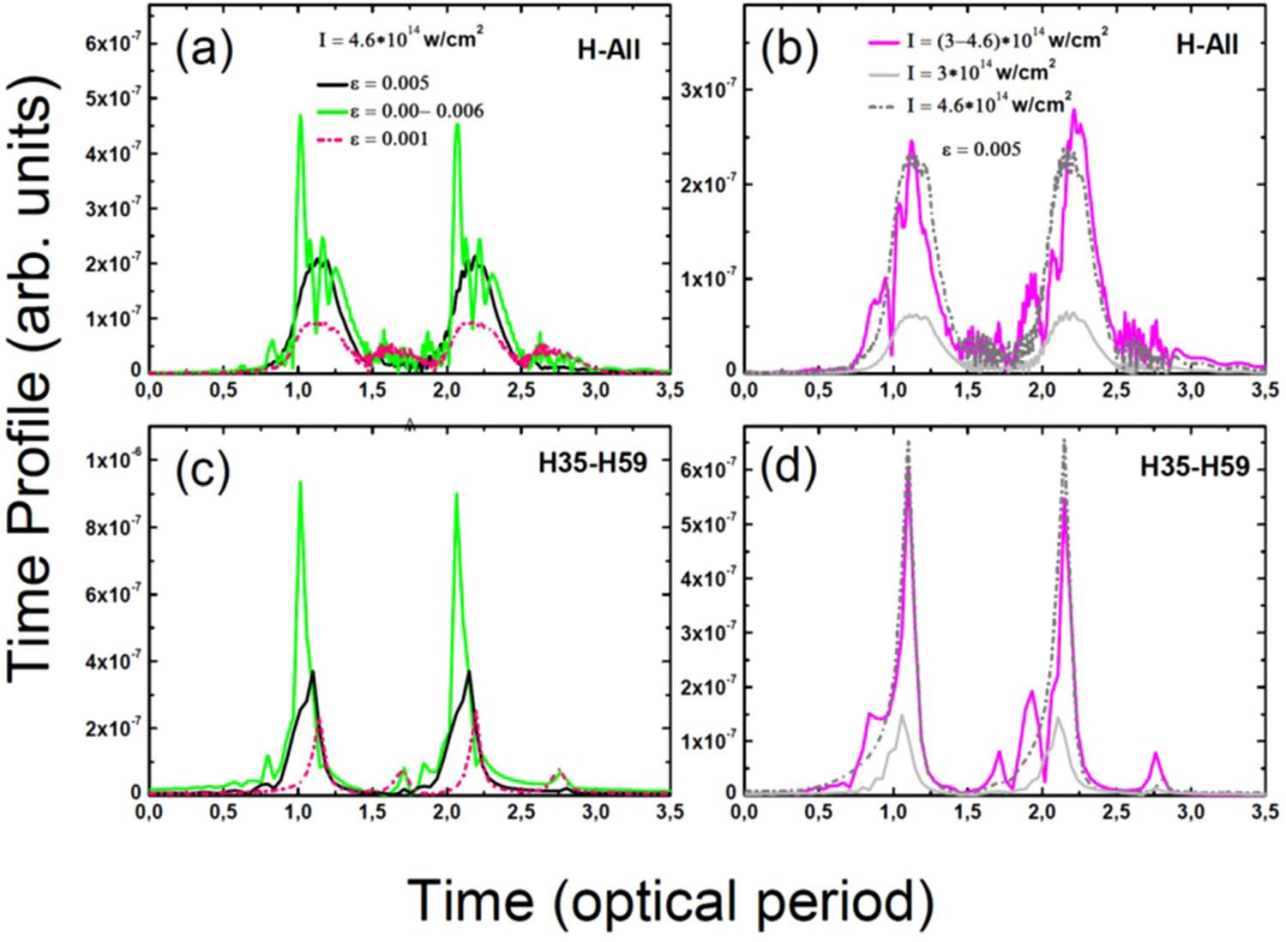
Train of pulses of harmonics emitted by a neon atom calculated from amplitudes and phases shown in Fig. 4. Panels (a and b) give train of pulse from all the harmonics and panels (c and d) show train of pulse from harmonics 35st to 59st. Red and black colors depict the cases when the atom exposed to a constant laser field (*I* = 4.10^14^ W*/*cm²) with *ε* = 0.001, *ε* = 0.005, respectively. We select here only the short trajectory. Green color present the case when intensity of the field is constant (*I* = 4.10^14^ W*/*cm²) and an inhomogeneity varying from *ε* = *0 to ε* = *0*.*006* with steps of 0.001. Dashed gray and solid gray colors show the case with constant inhomogeneity factor *ε* = *0*.*005* and intensities (*I* = 3.10^14^ W*/*cm²) and (I = 4.6.10^14^ W*/*cm²), respectively. Magenta color depict the train of pulses of harmonics calculated as the coherent sum of all spectra computed at constant inhomogeneity factor (*ε* = *0*.*005*) with field intensity from (*I* = 3.10^14^ W*/*cm²) to (I = 4.6.10^14^ W*/*cm²) with steps of (I = 0.1.10^14^ W*/*cm²).

Figure 5 compares the calculated train of emitted pulses by considering the near-field phase matching for the cases (a) with constant intensity (*I* = *4*.*10* ^*14*^ *W/cm* ^*2*^) and *ε* ranging from *0*.*001* to 0.006 and (b) with constant field inhomogeneity factor (*ε* = *0*.*003*) and intensity ranging from *I* = *3*.*10* ^*14*^ *W/cm* ^*2*^ to *I* = *4*.*6*.*10* ^*14*^ *W/cm* ^*2*^. Amplitude and phase are extracted from Fig. 4 considering two spectral windows: all harmonics (panels a and b) and harmonic 35 to 59 (c and d). Note that we take into account only the short trajectories in the calculations to obtain a single attosecond pulse per optical cycle. Indeed, due to different spatial and spectral properties, macroscopic (i.e., the far field) phase matching can select a single quantum path^48^. Short quantum path selection^42, 47^ would allow to further enhance the contrast of the main attosecond pulse as the long quantum path harmonic is out of phase (see Fig. 4c). In contrast to the inhomogeneous cases of *ε* = *0*.*001* with no near field phase matching (red curve), two bursts per cycle are emitted from all harmonics (Fig. 5a). The near field phase matching results look similar to the inhomogeneous case with (*I* = *4*.*10* ^*14*^ *W/cm* ^*2*^) and *ε* = 0.005 (black curve). However, the position of the peaks is slightly different and a high peak emission is observed. For harmonics selected between the 35^th^ and 59^th^ orders (Fig. 5c), these two peaks per cycle are more pronounced by a factor 3 compared to the case with *ε* = *0*,*005* and *I* = *4*.*10* ^*14*^ *W/cm²*. This is related to the fact that near field phase matching allows for constructive interferences between contributions from various regions of the plasmonic field with different field inhomogeneity factors.

## Conclusions

In this letter, we have shown that the attosecond emission from atoms immersed in a resonant plasmonic structure is strongly influenced by the strength of the laser induced plasmonic field. We show that the inhomogeneity of the electric field, within a cycle, breaks the symmetry and leads to the generation of both odd and even harmonics and two plateaus and cutoffs in the HHG spectra. The first plateau experiences a decrease in intensity while the second plateau is pushed towards higher harmonic orders compared to the homogeneous case. This is attributed, respectively, to either a destructive or constructive interference between the driving laser field and the plasmonic field. The emitted train of attosecond pulses is repeated every cycle of the infrared laser field rather than every half cycle of the laser field. Additionally, atoms experience various field strengths depending on their position in the resonant plasmonic field. Our calculations take this effect into account by calculating what we called the “near-field phase matching”. Our finding is that the coherent emission from atoms subjected to different field strengths and inhomogeneity can sum up to generate regular attosecond pulses. As a perspective, plasmonic fields have the potential to engineer the coherent emission of harmonics.

We have shown how HHG is sensitive to the local field. The harmonic electric field itself contains the imprint of the plasmonic field. An application would be to use the complete characterization of the harmonic spectral phase of the plasmonic enhanced HHG to access to the transition dipole moment over a large momentum space. This “self-probing” scheme or harmonic spectroscopy^49^ would allow to obtain spectral phase information on the electron dynamics and the local plasmonic field.

HHG assisted by plasmonic field in gases has not been clearly observed experimentally. However, we believe that our theoretical concept also stands for HHG attosecond control in solids assisted by field enhancement^20, 24^. The high density of atoms and the ease of nanofabrication make solid HHG a promising route.
